# Microarray experiments and factors which affect their reliability

**DOI:** 10.1186/s13062-015-0077-2

**Published:** 2015-09-03

**Authors:** Roman Jaksik, Marta Iwanaszko, Joanna Rzeszowska-Wolny, Marek Kimmel

**Affiliations:** Systems Biology Group, Faculty of Automatic Control, Electronics and Informatics, Silesian University of Technology, Gliwice, Poland; Department of Preventive Medicine, Northwestern University Feinberg School of Medicine, Chicago, IL USA; Department of Statistics, Rice University, Houston, TX USA

**Keywords:** Microarrays, Microarray pre-processing, Quality control, Transcriptome profiling, Measurement bias

## Abstract

Oligonucleotide microarrays belong to the basic tools of molecular biology and allow for simultaneous assessment of the expression level of thousands of genes. Analysis of microarray data is however very complex, requiring sophisticated methods to control for various factors that are inherent to the procedures used. In this article we describe the individual steps of a microarray experiment, highlighting important elements and factors that may affect the processes involved and that influence the interpretation of the results. Additionally, we describe methods that can be used to estimate the influence of these factors, and to control the way in which they affect the expression estimates. A comprehensive understanding of the experimental protocol used in a microarray experiment aids the interpretation of the obtained results. By describing known factors which affect expression estimates this article provides guidelines for appropriate quality control and pre-processing of the data, additionally applicable to other transcriptome analysis methods that utilize similar sample handling protocols.

**Reviewers:** This article was reviewed by Dr. Janet Siefert, Dr. Leonid Hanin, and Dr. I King Jordan.

## Introduction

Oligonucleotide microarrays belong to the most common tools used to describe changes in gene expression levels caused by altering the physical or chemical conditions. Microarrays can be also used to track differential expression patterns among various tissues and thus evaluate variability among individuals [1–3], they are used in SNP (single-nucleotide polymorphism) genotyping [4–7] and identification of transcription factor binding sites using the ChIP-chip (ChIP: chromatin immunoprecipitation) method [8–12]. Microarrays are also used to estimate genomic copy number using Comparative Genomic Hybridization (CGH) arrays [13–16] and in resequencing [17–22].

Microarray analysis offers a variety of methods allowing, among other, identification of genes which might be significant in a specific cellular response mechanism or a particular gene expression pattern that characterizes a particular disease. To obtain significant results, microarray data need to undergo statistical processing to differentiate between signal changes caused by direct experimental factors and arising from the indirect experimental factors such as specific methods used, as well as from inaccuracies of the measurements. This level of processing challenges led to studies of the compatibility of different microarray platforms [23–28] which usually is achieved by standardizing protocols and data analysis pipelines [29, 30]. Selection of an appropriate statistical method for microarray processing is a significant subject of scientific discussion and although microarrays have been in use for more than fifteen years, many issues related to data analysis remain unresolved.

The most discussed issues concern the algorithms used for the data normalization [31, 32], whose goal is to eliminate differences between samples that originate from technical aspects of the microarray handling which may confound the biological differences in a given experimental setup. A similar goal underlies methods used for batch-effect removal, a step which is crucial when comparing datasets that originate from different times and laboratories [33]. Other frequently-discussed issues concern the identification of sample differentiating genes [34, 35] and evaluation of noise level in the sample [36], as well as methods to evaluate contamination or damage on the microarray’s surface [37, 38]. The most commonly used microarrays, produced by Affymetrix, are known for additional issues related to their particular design which influence the final results. These include problems resulting from several measurements of expression level for a single gene [39, 40], incorrect assignments of probes to genes [41, 42], incorrect evaluation of the background level and non-specific probe hybridization signals [43], and the effects of distinct probe features on data processing algorithms [44].

The most significant disadvantages of microarrays include the high cost of a single experiment, the large number of probe designs based on sequences of low-specificity, as well as the lack of control over the pool of analyzed transcripts since most of the commonly used microarray platforms utilize only one set of probes designed by the manufacturer. Other weaknesses of microarrays are their relatively low accuracy, precision and specificity [45] as well as the high sensitivity of the experimental setup to variations in hybridization temperature [46], the purity and degradation rate of genetic material [47], and the amplification process [48] which, together with other factors, may impact the estimates of gene expression.

## Review

### Microarray structure

**Fig. 1:**
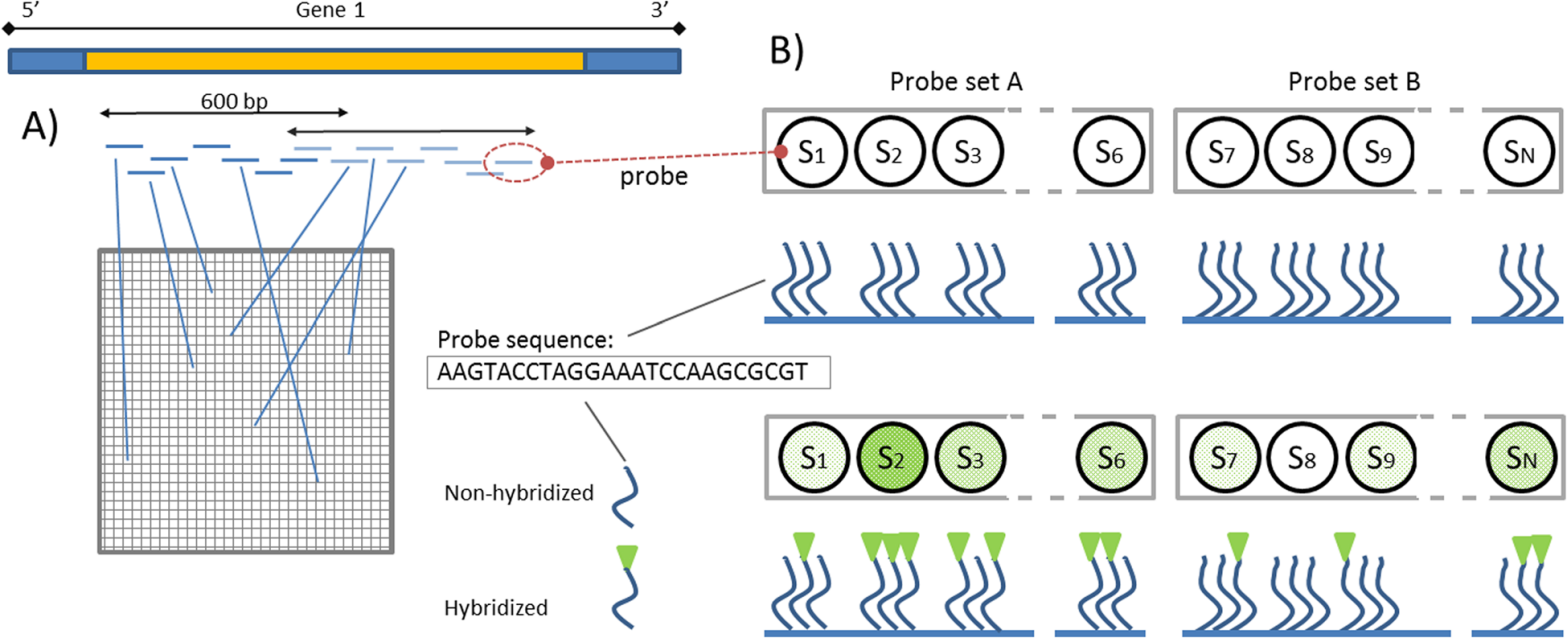
Microarray schematics. **a** Probes corresponding to the characteristic fragments of a given gene are placed in different locations across the array; **b** Single probes are arranged in sets corresponding to the same region of the gene. DNA that hybridizes to the probe can be detected using a fluorescent reporter system. Increasing the number of probes to which cDNA hybridizes correctly, increases the contrast between this probe set (*probe set A*) and any other probe set (*probe set B*)

A typical microarray consists of oligonucleotides which are several dozen nucleotides (nt) long attached to the surface of a glass slide. Using appropriate photolithographic masks, a single nucleotide A, C, T, or G is attached at a time, and therefore it is possible to construct a microarray with hundreds of thousands of different oligonucleotide sequences which are complementary to characteristic fragments of known DNA or RNA sequences. These characteristic fragments are arranged in sets called probes [49]. A sample containing DNA or RNA molecules is spread on the surface of a microarray and its components hybridize specifically with their complementary probes, which are located in multiple copies across the microarray (Fig. 1). The amount of material hybridized to a given probe is determined by a fluorescence-based method and although the relationship is not linear the fluorescence intensity reflects the amount of DNA or RNA of a given gene in the sample [50]. This approach allows quantifying the level of transcripts of thousands of genes in a relatively short time.

The most widespread microarray is the Affymetrix 3′IVT (3′ *in vitro* transcription), i.e. HG-U133A or HG-U133_Plus_2, which is assembled as 11 sets of perfect match (PM) probes consisting of 25 nt sequences, which in most cases were chosen out of 600 nt sequence fragments located near the 3′ end of a specific transcript. For every PM probe on the microarray, a MM (mismatch) probe exists in which all nucleotides but one are identical to those on the corresponding PM probe but the original 13th nucleotide is replaced by a non-complementary one. The rationale behind the MM probes is to gauge the level of nonspecific hybridization [51], although the usefulness of this concept has been doubted (see further on).

The most recent generation of Affymetrix microarrays, such as the HuGene 1.0ST, is constructed using probes similar to the standard PM probes but with affinity not to the noncoding part of the 3′ end but rather to the individual exons in a given transcript. In this design the MM probes are replaced by the Background Intensity Probes (BGP), which are designed to evaluate background intensity levels for probes of different sequence characteristics. BGP are a set of about 1000 probes, non-complementary to any human gene sequence, with a variable ratio of GC nucleotides in the sequence. This approach enables a better evaluation of non-specific hybridization across the microarray compared with MM probes, for which the signal often exceeds the PM signal due to probe-specific effects [52]. Additionally, lowering the number of probes which evaluate non-specific hybridization allows inserting of a much higher number of PM probes. The probe set in the new generation of whole transcript microarrays is constructed with two levels, exon and gene level. The exon probe set includes 4 probes on average, which are tailored for individual exons, and then these are clustered, usually in groups of around 25, creating sets for individual genes. Using this approach it is possible to determine levels of individual differently-spliced transcripts.

Another popular system is the Agilent microarray platform which was built using the SurePrint technology that allows using considerably longer, 60 nt-long probes. While probes are longer than in the Affymetrix system, the number of probes per gene is considerably lower, 8 on average in the most expensive set of exon microarrays (2 × 400 k) or 2 in the least expensive platform (8 × 60 k). As the Agilent probes are longer than those in the Affymetrix microarrays, the system tends to be more specific which is an obvious advantage, but on the other hand the lower number of probes per gene makes Agilent microarrays more sensitive to single nucleotide variations. These latter should not affect the signal if they result from amplification errors [53], but they may influence the expression estimates resulting from characteristic features of the sample analyzed. In the case of the Affymetrix microarray system these sources of error will only have a minor impact, as they influence signal only in an individual probe for a transcript or a transcript-specific probe-set. Single nucleotide polymorphisms do not block the hybridization but lower its efficiency, which can be interpreted as a significant decrease of gene expression, a feature which is used to estimate the level of nonspecific hybridization using mismatch probes [54, 55] or to assess allelic frequencies using SNP microarrays [56]. In the Affymetrix systems the signal from one badly designed probe, which may be based on inaccurate data from a sequence database, can be easily eliminated from further analysis [41] without significant decrease in the precision of gene expression estimate, while in the Agilent systems the same design glitch might cause significant difficulties in the evaluation of gene expression levels.

Microarrays provide expression data for thousands of genes, but platform differences contribute to low accuracy of microarrays and for this reason they are only used to identify potentially significant genes in the experimental conditions studied. Precise assessment of the expression level of these presumably significant genes requires additional studies using more accurate methods such as real-time PCR (polymerase chain reaction) which, in turn, are not suitable for large-scale analyses. However, some steps of the microarray protocol are shared by the validation methods, affecting data quality in a similar manner.

### Biological background of microarray experiments

**Fig. 2:**
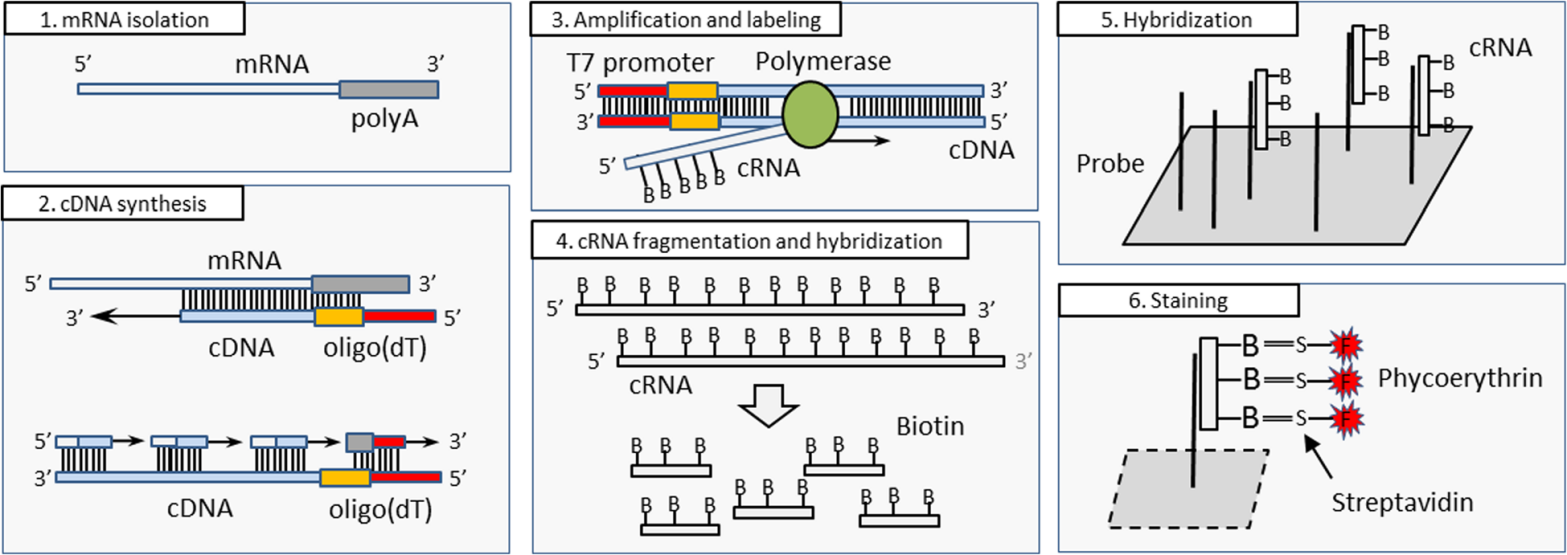
Individual steps of a microarray experiment. After isolation of sample mRNA (1) synthesis of cDNA (complementary DNA, step) chains begins with addition of oligo(dT) primers (2), then cDNA is amplified, producing cRNA (complementary RNA), which is labelled with biotin (3) and later fragmented (4). After such preparation sample cRNA is ready for hybridization with microarray probes (5) and ready for the final staining process (6)

The microarray experiment is a multi-stage process in which the accuracy of each individual step may influence the gene expression estimates. Precise understanding of each step is very important not only for the experimenter but also for the person performing data pre-processing. In order to avoid mistakes that occur during the experiment, its accuracy and the condition of the biological material are controlled in various steps, as shown in Fig. 2. The procedure used in a microarray experiment is very similar across different platforms, and therefore for simplicity the following description is based on the procedure used for the Affymetrix microarrays.

#### Step I: RNA isolation

**Fig. 3:**
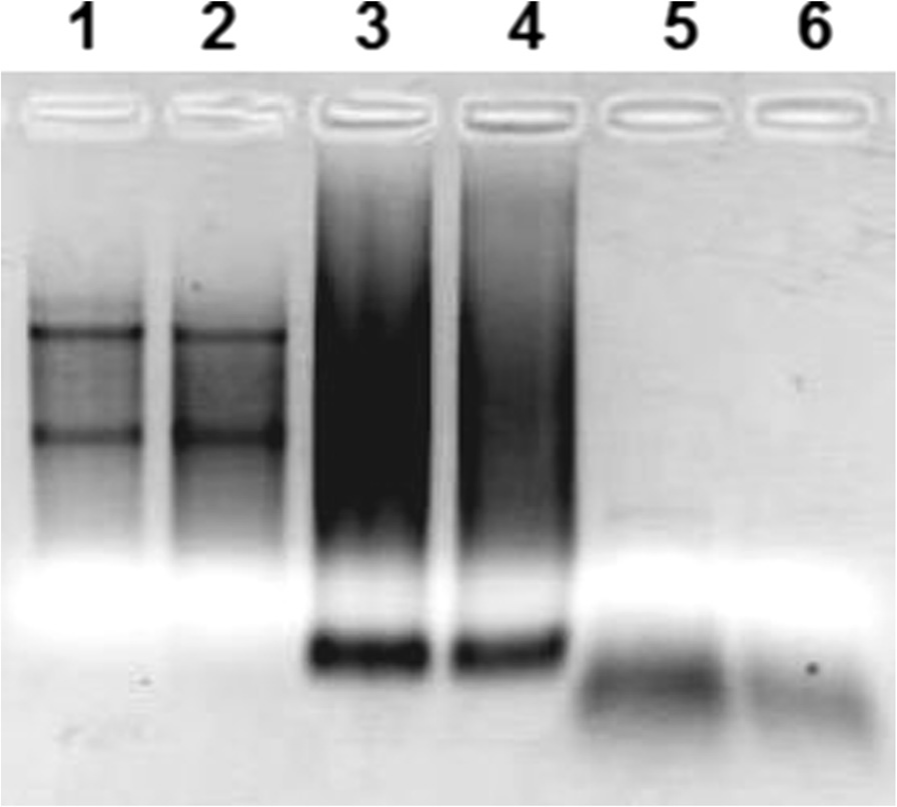
Electrophoretic analysis of the products obtained at various stages of a microarray experiment. Lanes 1 and 2, isolated RNA; 3 and 4, purified cRNA; 5 and 6, fragmented cRNA (Image courtesy of Herok R. - unpublished data)

In the first step RNA is isolated from the cells and its concentration and extent of degradation is controlled by the use of a spectrophotometer (quantity) and a bioanalyzer (quality). In a high-quality RNA sample ribosomal RNA (rRNA) constitutes over 80 % of the entire RNA, and despite the fact that it is rarely a target of a study in fields other than bacteriology and phylogenetics, its concentration is a good indicator of the overall RNA quality, both before and after the experiment. Prior to hybridization the extent of RNA degradation can be assessed by RNA electrophoresis or a bioanalyzer using the RNA integrity number (RIN) as a benchmark [57]. Fig. 3 shows an example of an image made after electrophoresis in agarose gel. The first two lanes show RNA directly after isolation. The two most distinguishable bands correspond to the 18 and 28S rRNA and their absence would indicate that RNA were highly degraded [58].

RNA quality can also be evaluated after the experiment by analyzing results from a specific group of control probe-sets (as described in Table 1) designed to target certain housekeeping genes (group 1) or rRNA (group 2). As with other probe-sets a single expression intensity is however non-informative since its value, expressed in arbitrary units, depends on the characteristics of the sample [59], the experimental conditions, such as for example the ozone level in the laboratory [60], and the data pre-processing methods used [61]. For this reason arbitrary criteria based solely on single expression intensities are usually ineffective and a comparison between arrays or between probe-sets is required.

**Table 1:** Reference genes found on a typical Affymetrix 3’IVT microarray. Amplification and hybridization control RNAs are added in various proportions and quantities as indicated in the last column. The amplification control transcripts are added using various dilutions which results in an estimated copy numbers ranging from one copy per 6,667 to 100,000 transcripts in the studied RNA sample. The hybridization control consists of biotinylated and fragmented cRNAs added in various amounts that result in a final concentrations ranging from 1.5 to 100 pM

Group	Type	Name	Description
1	Housekeeping genes	AFFX-HSAC07/X00351	ACTB - β-actin gene responsible for the structure of the cell
		AFFX-HUMGAPDH/M33197	GAPDH – enzyme which takes part in glycolysis
		AFFX-HUMISGF3A/M97935	STAT1 – transcription factor
2	Ribosomal RNA	AFFX-HUMRGE/M10098	Gene coding for 18S rRNA subunit
		AFFX-M27830	Gene coding for 28S rRNA subunit
		AFFX-r2-Hs18SrRNA	Gene coding for 18S rRNA subunit - version 2
		AFFX-r2-Hs28SrRNA	Gene coding for 28S rRNA subunit - version 2
3	Amplification control (Poly-A spike)	AFFX-DapX / AFFX-r2-Bs-dap	Dap gene of *B.Subtilis* bacteria - proportions 1:6,667
		AFFX-ThrX / AFFX-r2-Bs-thr	Thr gene of *B.Subtilis* bacteria - proportions 1:25,000
		AFFX-PheX / AFFX-r2-Bs-phe	Phe gene of *B.Subtilis* bacteria - proportions 1:50:000
		AFFX-LysX / AFFX-r2-Bs-lys	Lys gene of *B.Subtilis* bacteria - proportions 1:100,000
4	Hybridization control (Bacterial spike)	AFFX-BioB / AFFX-r2-Ec-bioB	BioB gene of *E.Coli* bacteria – quantity 1.5 pM
		AFFX-BioC / AFFX-r2-Ec-bioC	BioC gene of *E.Coli* bacteria – quantity 5 pM
		AFFX-BioDn / AFFX-r2-Ec-bioD	BioD gene of *E.Coli* bacteria – quantity 25 pM
		AFFX-CreX / AFFX-r2-P1-cre	Cre gene of P1 bacteriophage – quantity 100 pM

Each of the control probe-sets exists in three variants, each targeting a different region of the selected transcript - its central section and the 3′- and 5′-ends. This allows assessing the degradation rate of individual transcripts by examining the 3′/5′ probe-set signal ratios, which can be compared to the threshold defined by the manufacturer and ratios obtained for other microarrays, in order to assess the homogeneity of degradation level across individual samples. In order to aid the assessment of post-experimental RNA degradation, more complex methods have been developed including RNA degradation plots [62] or mixed effect models based on individual probe and transcript characteristics [63].

#### Step II: cDNA synthesis

**Fig. 4:**
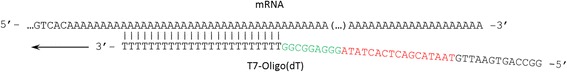
cDNA synthesis based on a commercial T7-oligo (dT) primer. The arrow indicates the direction of synthesis, red font indicates the promoter sequence used in the amplification. The green region is the sequence spacer that separates the primer from the (T)_24_ motif

At the beginning of this stage external RNA controls (ERCs) are added which serve as a control of cDNA synthesis independently of the volume and condition of the input material. For this purpose bacterial RNA is used (the so-called poly-A spike) with no homology to known human genes. Following this, the cDNA synthesis process is performed by the use of oligo-dT (a primer with a short sequence of deoxy-thymine nucleotides) or random primers. Oligo-dT binds to the poly-A tails of mRNAs, initiating the synthesis of the complementary strand in a process of reverse transcription (Fig. 4). This process does not work for rRNA molecules since unlike mRNA molecules they do not have a poly-A tail, and for this reason it is not necessary to remove the rRNA prior to this process. However, in some cases rRNA can be polyadenylated in human cells [64], and conversely, not all mRNAs have a poly-A tail, and there are also reports of mRNAs that exist in two forms both polyadenylated and non-polyadenylated [65].

The second strand of the cDNA is then created by using the first strand as a template. Addition of ribonuclease causes RNA cleavage at nonspecific sites, leaving only short fragments attached to the cDNA (Fig. 2). These fragments are then used as primers for the polymerase which synthesizes the second strand of the cDNA, removing the remaining mRNA fragments found on its way. Measurement of cDNA concentration, which allows standardizing it across various samples, is not a part of the standard experimental procedure for eukaryotic cells, due to the presence of other nucleic acid species that affect the spectrophotometric measurement, whose removal requires additional cDNA purification. This step is strongly influenced by any previous RNA degradation, which leads to the creation of truncated mRNAs (from the 5′-end) [66]. When oligo-dT primers are used during cDNA synthesis these truncated mRNAs are read from the 3′-end only to the position of truncation, and the remaining part is lost due to the lack of poly-A. In such a situation probes located further from the 3′-end usually show lower signal intensity, a phenomenon which is the basis of RNA degradation plots used to assess the mRNA quality [62]. In order to reduce this effect, on 3′IVT microarrays probes from a single set are selected based on a very small region of 600 bp located close to the 3′-end of the mRNA. To further reduce this bias sophisticated methods have been developed that take into account the location of regions targeted by probes in order to correct the signal intensities [67, 68].

The 3′-end bias does not occur when random primers are used for the cDNA synthesis. Random primers do not require a poly-A tail since they can attach to any region of the mRNA and not only to its 3′-end, promoting synthesis in a 3′ ➔ 5′ direction, and a very strong 5′-end bias can be observed as shown in ref. [69].

Although many of the available cDNA synthesis kits include a combination of oligo-dT and random primers, kits based solely on oligo-dT are commonly used especially for the 3′-IVT platform where 3′-UTR sequences are of the highest importance since they are targeted by oligonucleotide probes.

Oligo-dT-based cRNA synthesis introduces an additional bias that may affect the results of a microarray experiment. First of all, because of the mRNA degradation problem, oligo-dT primers are a good choice only if the region of interest is located in the vicinity of the 3′-UTR, since large distances between the region targeted by probes and the poly-A can decrease the precision of expression level estimates [70]. If the analysis requires the entire transcript as in the case of WT (whole-transcript) microarray platforms where individual exons are analyzed, random primers are required. Additionally, oligo-dT is assumed to bind only to the poly-A tail of the transcript, requiring a long continuous strand of A nucleotides, as shown in Fig. 2. However, partial primer complementarity (i.e. complementarity of only 8 adenine nucleotides in the primer’s sequence) is sufficient for the reaction initiation, and due to the random nature of the attachment it can also bind to the A-strands found commonly in the UTRs [71]. Further, with increasing concentration of oligo-dT the chance of attaching multiple oligonucleotides to a single mRNA are increased. In such situation the synthesis may start from two distinct regions but the reaction located closer to the 3′-end might be blocked by the second reaction, again producing truncated cDNA products [71]. This phenomenon can therefore affect the entire probe-set signal intensity of the targeted transcript if its sequence includes simple repeats built predominantly of A nucleotides.

#### Step III: Amplification and labeling

In this step the newly-synthesized cDNA is replicated (amplified) in a process of *in vitro* transcription. The goal of this step is to obtain a large quantity of cRNA containing biotinylated C and U nucleotides that will be required in the subsequent steps [58]. For this purpose another fragment of oligo-dT is used, marked in red in Fig. 2, which serves as a promoter for the T7 bacteriophage polymerase.

The efficiency of this reaction and its consistency between samples has a decisive impact on the final experimental outcomes [72]. There are many factors which influence the efficiency of this reaction including the structural properties of the cDNA itself which, depending on the GC content, can affect the efficiency of the polymerase [73] and form secondary structures [74]. This step is completed with a cleanup and quantification of the cRNA which allows for control of the total reaction yield and purity of the sample. The product of the amplification reaction can be observed in lanes three and four of the electrophoresis gel (Fig. 3). rRNA is no longer visible, and due to the variability in length of the cRNAs there are no easily distinguishable bands visible on the gel.

Post-experimental control of cRNA level variations, utilizes the signals of probes targeting a reference RNA (poly-A spike) added prior to cDNA synthesis and signals of housekeeping genes which should be on a similar level across all samples. The poly-A spike contains transcripts of five *B. subtilis* genes (Dap, Lys, Phe, Thr, and Trp) which are added in various proportions to the isolated RNA. Since they all include a poly-A tail they undergo the same procedure as the RNA analyzed, independently of its condition. Lys gene RNA is added at the lowest concentration (1:100,000 of the total RNA) which is close to the sensitivity level of the microarray. Its detection in at least half of the microarrays of a given experiment is a good indicator of a properly conducted procedure. The remaining reference RNAs are added in increasing concentrations Lys < Phe < Thr < Dap with Dap being the highest and close to the probe signal intensity saturation level.

The amplification products no longer have the T7 promoter, although the spacer sequence between the promoter and the (T)_24_ primer (green in Fig. 3) is also amplified [75]. Since this fragment is copied with each cRNA its quantity is very large, and since it can bind to probes having a similar sequence it might affect their signal intensity [69]. It is believed that the process of amplification might be the source of inconsistent signals among samples, as it depends highly on the experiment conditions and the transcript structure [74, 76], becoming the main motivation for the development of microarray protocols that do not require RNA amplification [77].

#### Step IV and V: cRNA fragmentation and hybridization

cRNAs obtained in the previous step are cut into 50–100 nt fragments shown in lanes five and six of the electrophoresis gel (Fig. 3). After this, another set of external RNA controls (ERCs) that originates from P1 bacteriophage and *E. coli* bacteria (termed bacterial spikes) is added to the RNA pool. Similarly to the poly-A spike, bacterial RNA is added in various concentrations with the following relations satisfied: bioB < bioC < bioD < Cre (group 4 in Table 1). BioB, bioC and bioD originate from the *E. coli* genes used in the synthesis of biotin, while Cre is isolated from P1 bacteriophage where its gene product serves as a recombinase [78]. This bacterial spike is already converted to cRNA and fragmented allowing to control the hybridization process, independently of the efficiency of labeling and amplification used in the previous steps to obtain cRNA [58]. After this the mixture of various cRNAs is transferred on to the microarray chip, initiating the hybridization process.

Hybridization is the most time-consuming step of the entire microarray procedure. During approximately 16 h, in which microarrays are incubated in a hybridization oven set to 45 °C, the cRNA binds to the specific probes attached to the glass surface of the microarray chip. The dynamics of the hybridization process depends on many factors which, as in the amplification step, depend on both the reaction conditions and structural properties of the individual cRNA molecules which may significantly affect the experimental outcomes [79, 80]. Prolonged hybridization can cause sample drying and uneven distribution of the material on the surface of the chip. Additionally, evaporation of some of the water can change the salt concentration in the buffers and significantly affect the efficiency of the process [81].

The main purpose of the bacterial spikes added before the hybridization step is to control the consistency of hybridization conditions across all samples, assessing the overall microarray performance [82]. Flaws in the experimental procedure cause either variations in expression intensity range or in the relations among individual bioB, bioC, bioD and Cre transcripts, although one has to remember that flaws in the hybridization process affect other transcripts as well. For this reason, hybridization inconsistencies should be also visible in probe-sets targeting other cRNAs, including the poly-A spike controls. If variations are only present in the bacterial spikes, the problem most likely originates from inaccuracies in their preparation or their concentration in the pre-hybridized cRNA. All of the possible scenarios for housekeeping genes, poly-A, and bacterial spike controls are summarized in Table 2.

**Table 2:** Problems detected by different control probe-sets and their possible reasons^a^

Housekeeping genes	Poly-A spike	Bacterial spike	Possible reason
error	ok	ok	Poor quality of the mRNA analyzed
error	error	ok	Problems during amplification/labeling
error	error	error	Problems during hybridization/washing
ok	ok	error	Inaccurate preparation of bacterial spike
ok	error	ok	Inaccurate preparation of bacterial poly-A spike

^a^Other possible combinations of errors rarely occur in practice

Bacterial spike controls are a good indicator of problems that may occur during the hybridization procedure, although they fail to detect uneven hybridization, since the probe-set intensity is obtained after summarizing signals of over 20 individual probes, spread over the entire surface of the microarray (3′IVT arrays) or located in a small region at the middle of the array (WT arrays). For this purpose the quality control of each sample should include the analysis of an image of the microarray surface, which is either a complete scan saved in a DAT file, or more commonly a recreated image based on the individual probe intensities stored in a CEL file [83, 84].

The main assumption made in design of a microarray is that probes targeting a single transcript are placed randomly on its surface. For this reason, variations in the signal intensity of specific regions suggest reasons other than the biological variation between the analyzed mRNAs. Such differences among regions, termed image artifacts, are mostly caused by bubbles of air or small levels of impurities, which were added into the microarray cartridge with the experimental solutions [85]. Such artifacts appear very commonly, although they usually have a very small size and are handled efficiently by summarization methods, which are insensitive to a small number of outlying values. The main problem occurs when the artifact covers a significant percentage of the array surface or its intensity is extremely high and close to the saturation level of the probes. Such artifacts are mainly caused by uneven hybridization and affect not only the expression estimates from probes located in its region, but also the remaining probe signals. This latter effect is due to data processing, which utilizes expression levels of all or of a significant fraction of the probes on the microarray [38].

Microarray surface artifacts can be visualized by either creating an image, based on single probe expression intensities in a convenient (usually logarithmic) scale, or by analyzing differential images created by subtracting the signal of each probe on a single microarray from that on another reference array created by, for example, calculating the median intensity level of each probe across all microarrays in a single experiment [37]. If a defective array is found, probes affected by an aberration may be separated and removed from the subsequent data analysis or even recreated using imputation techniques [38, 37, 85, 86]. Microarrays affected by a very large aberration should be removed from the study, as they no longer serve as a reliable source of information.

#### Step VI: Washing and staining

Washing follows the cRNA hybridization and is used to remove cRNA non-specifically bound to the microarray surface. Again, in this step small variations in the reaction conditions may affect the expression estimates [87]. Depending on the conditions of the washing process (temperature, salt concentration, calcium and magnesium ion levels in the buffer) non-specifically bound cRNA is removed with varying efficiency, affecting the sensitivity and background level of the entire microarray. The binding strength of cRNAs depends not only on their complementarity level but also on the temperature of the hybridization and their sequence characteristics, mainly the GC content [88] and specific base positions inside the sequence [44]. Separation between the binding strength of non-specifically bound GC-rich cRNA and GC-poor cRNA with perfect complementarity is not very sharp, affecting the final intensity level of cRNAs depending on their sequence characteristics [50], which can be only reduced using sequence-based normalization approaches during the data pre-processing step [89, 90].

The washing process is followed by staining of the hybridized cRNA using a streptavidin-phycoerythrin complex (Fig. 2). Streptavidin is a protein with high binding affinity to the biotinylated nucleotides used in the cRNA preparation, while phycoerythrin is a fluorescent dye used for quantitation of the hybridized cRNA. The quality of the fluorophore used significantly affects the fluorescence intensity of the microarray, decreasing its sensitivity if it is exposed for too long to daylight [91].

#### Step VII: Scanning

In this step the microarray cartridge is placed in the microarray scanner where the fluorescence of the phycoerythrin bound to the cRNA is excited using a laser. The level of fluorescence is measured by the scanner’s detector and is assumed to be proportional to the amount of cRNA bound to the corresponding probe. The length of this process depends on the size of the microarray and in most cases lasts around 10 min for a single array. During the scanning process all arrays are placed inside the scanner’s chamber so that the fluorescence intensity is not affected by differences in the length of exposure to daylight, which could increase the differences among microarrays in both the scale of the measurements and the sensitivity level. It is advised to scan each microarray only once, since each subsequent scan decreases the fluorescence intensity by 10–20 %, due to decay of the fluorophore [92]. The fluorescence intensity of cyanine-based dyes also used in microarray experiments, such as Cy5, can be further affected by the ozone concentration in the laboratory, a factor which is both time- and location-dependent, and can become a major source of among-experiment inconsistencies [60, 93].

#### Step VIII: Data pre-processing

The last stage involves data pre-processing which starts by analyzing the microarray image stored in the DAT file, whose goal is to obtain single fluorescence intensity for each probe based on the 16 pixels of the original microarray image. This step is performed by the Affymetrix software and returns a CEL file as an output, in which each probe, at a specific position on the microarray, has a signal intensity assigned to it. These individual probe intensities are used in the subsequent preprocessing steps, during which each array is standardized by first estimating and then subtracting the background signal in order to reduce the effect of non-specific hybridization [44]. Following step is to perform normalization procedure which reduces the differences in probe intensities that originate from differences in experimental conditions and cRNA concentration [31, 94]. The final step of pre-processing is the summarization, in which a single expression estimate is calculated for each probe-set based on the intensity of the individual probe signals [95]. Summarization step is highly dependent on the quality of the probe and probeset definitions which are in many cases low due to inaccurate transcriptome data at the time of microarray design. This can result in probesets targeting transcripts of multiple genes due to low probe specificity, probes that do not map any of the known transcripts [41, 42] or multiple probesets that map the same gene [39, 40], requiring the development of methods used for the validation of existing probes and for probeset redefinition [41, 42, 96].

Selection of the pre-processing strategy can have a very large impact on the experimental outcomes [94] and often requires a few assumptions which are not always acceptable. The main assumption made by pre-processing methods is that the total level of mRNA in the cell does not vary significantly among samples, regardless of the experimental conditions and cell lines used. This assumption is required for the standardization approaches based on mean and median scaling or more complex approaches, such as quantile normalization [31], and its natural consequence is that the amount of differentially-expressed features with increased or decreased levels will be always similar. For example, in the case of global transcript level changes in cells with inhibited transcription, one might expect to detect predominantly transcript down-regulation, whereas after applying quantile normalization it is very probable that a significant number of up-regulated transcripts will be observed, due to intensity distribution transformations.

Another important assumption is forced by the massively parallel experimentation of the microarray technique which allows for assessing expression level of thousands of genes simultaneously. We have to assume that the reaction conditions for each individual gene were similar while knowing that due to various molecular properties of the analyzed RNA/DNA fragments it is impossible to properly optimize each of the individual reactions. Most of the data processing methods make this assumption although some standardization methods also exist that utilize probe and RNA/DNA sequence information in order to reduce the signal differences resulting from sub-optimal amplification and hybridization conditions that affect gene expression estimates to a varying degree [89, 90].

## Conclusions

Despite successful studies of reproducibility [27] and specificity [97], microarrays have been often subject of criticism as a method which fails to identify relevant information that can be transferred directly into clinical applications [98]. The main reason is that statistical significance often differs from biological relevance due to a very limited number of samples or to the influence of other factors, such as cellular heterogeneity or variability of the morphological features, which are difficult to separate from the studied features. This highlights the importance of experimental design which utilizes an adequate number of samples and biological replicates to answer questions defined in the project.

**Fig. 5:**
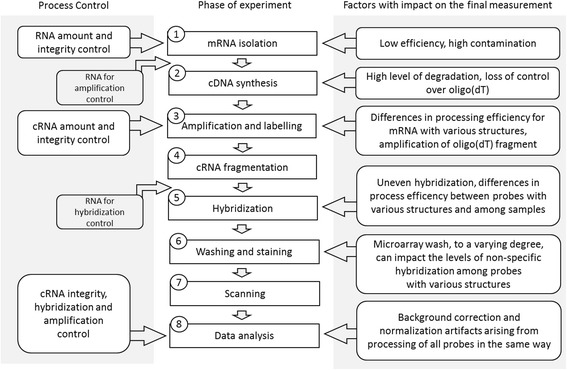
Individual steps of a microarray experiment. Right panel describes factors that may affect experimental outcomes, left panel typical methods that are used to validate each of the processing steps

In this article we describe the experimental protocol used for Affymetrix microarrays and important factors that may influence its outcomes, summarized in Fig. 5. The entire procedure has been subject of many changes since the first Affymetrix microarrays were released, mainly involving different sets of reagents which allow obtaining a higher yield of reactions with shorter incubation times. The most important changes include the transition from one-step to two-step cDNA synthesis in 2004, and the addition of whole transcript (WT) microarrays in 2009, which utilize different sets of reagents produced by Ambion. Apart from the differences in cDNA synthesis, which as described in step two of the experimental protocol, is based on random primers instead of oligo-dT, significant modification was also made to the labeling process of WT microarrays., The labeling takes place after cDNA fragmentation and uses terminal deoxynucleotidyl transferase (TdT) that adds labeled nucleotides only at the 3′-end of the cRNA. Despite similar methods of oligonucleotide probe design, changes in the experimental procedure might explain the differences in transcript level estimates obtained using various platforms, and their modifications with time might be a source of the inconsistencies observed among experiments conducted by different laboratories and even in the same laboratory, when separated by long periods of time.

The capabilities of microarray studies are limited, since the measurement of transcript levels provides only a rough estimate of the intracellular conditions at a specific time point, and is affected by a plethora of experiment-specific factors. The process of discovery of new drugs, using expression or genotyping microarrays, is therefore uneven in pace and in some cases might be even misleading. However, microarrays can be successfully used to validate the effects of existing drugs by helping to identify their targets and off-target effects [99]. Microarrays are becoming less popular due to the decreasing costs of the RNA-seq methods [100], although one has to remember that some of the steps used in the microarray procedure, with their drawbacks and limitations, are also utilized in other techniques including the RNA-seq approaches [101–103]. Despite the evolution of experimental procedures, the fundamental principles behind microarray experiments remain similar and their understanding is also an essential step towards appropriate interpretation of the data provided by more advanced but related methods.

## Reviewers’ comments

### Reviewer’s report 1: Dr. Janet Siefert

Jaksik et al. have written a review article on the use of microarrays and the cautions and complications of using the results of them to evaluate research data. The Kimmel lab has considerable experience, over several years, with microarray data so the expertise of his team to evaluate and write such an article is well placed. I find their review to be comprehensive and thorough. It will be of considerable use to anyone considering employing microarray experiments as well as those who need to troubleshoot previous use of microarrays and accompanying statistical evaluations. Although the published literature offers a number of articles reviewing microarray use, it is the expertise of this team, as statisticians actively working with numerous microarray data sets, that makes this article valuable to the researching community.

### Reviewer’s report 2: Dr. Leonid Hanin

This is an interesting article that lists and analyzes in detail various sources of errors and inconsistencies associated with microarray technology. It represents an important step towards answering the following fundamental question: Is microarray technology a reliable tool for furthering our understanding of biological systems at the genomic level or is it bound to produce a lot of biological/technological artefacts and largely generate false knowledge? I believe the main deficiency of the article is that it is entirely qualitative in that no quantitative estimates of the impact of various factors identified in this work or of their relative importance were given. The basic question that a researcher utilizing microarrays would ask is whether impact of these factors is minor or major. The article does not provide any information or opinion in this regard. Given that the processes collectively forming microarray technology are either biochemical or physical in nature, estimating the effects of various factors on gene expression signals quantitatively seems to be in principle possible. For example, here are two relevant publications, just from the top of my head, about the physics of DNA/RNA hybridization, see also references therein:

1. E. Carlon, T. Heim (2006), Thermodynamics of RNA/DNA hybridization in high-density oligonucleotide microarrays, Physica A 362: 433–449.

2. A. Ferrantini, E. Carlon (2008), On the relationship between perfect matches and mismatches in Affymetrix Genechips, Gene 422: 1–6.

On a more technical level, I have the following questions and comments.

1. One of the sources of uncertainty in determination of gene expression levels that was not mentioned in the article is errors in gene finding. While for well-annotated genomes of model organisms they are probably insignificant, for many other organisms type I and II errors in gene finding may be as high as 10–15 %.

*Authors’ response: Description of probe design flaws resulting from inaccurate transcriptome data were added to the description of the data pre-processing step.*

“Summarization step is highly dependent on the quality of the probe and probeset definitions which are in many cases low due to inaccurate transcriptome data at the time of microarray design. This can result in probesets targeting transcripts of multiple genes due to low probe specificity, probes that do not map any of the known transcripts [41, 42] or multiple probesets that map the same gene [39, 40], requiring the development of methods used for the validation of existing probes and for probeset redefinition [41, 42, 96]. “

2. Another factor that was mentioned only very briefly in the text but probably deserves more discussion is heterogeneity of biological material from which mRNA is extracted. It may include different types of cells, cells in various phases of their life cycle, quiescent and proliferating cells, etc.

*Authors’ response: Cellular heterogeneity is a major problem in many biological studies and can indeed significantly affect the results of a microarray study. However because this problem is unrelated to the technical aspects of the microarray protocol, we find it to be outside of the scope of this article.*

3. It was mentioned, again very briefly, in the Conclusions section that utilizing several replications may improve design of microarray experiments. I think this is a very important point. I find it pretty appalling that a lot of microarray experiments with so many sources of variation and error were based on a single run of the process! What minimum replication number would the authors of the article recommend?

*Authors’ response: Based on our experience we suggest using at least 3 replicates for studies based on cell lines. From a statistical point of view a minimum of 3 samples are required for the estimate of standard deviation to be valid. Higher number of replicates might be highly beneficial for experiments dealing with poor quality material or studies aiming to detect small differences in gene expression. For experiments based on samples extracted from multiple patients, replicates are usually not necessary since the confidence level increases with the number of patients studied.*

4. It was stated in the Microarray Structure section that the “amount of material hybridized to a given probe… is related to the amount of DNA or RNA of a given gene in the sample. ”What is this functional relationship? Is the magnitude of the optical signal produced by microarray chip proportional to the copy number of a gene’s RNA transcript in the sample? What happens to this relationship when microarray output data are pre-processed?

*Authors’ response: The fluorescence intensity of probe is proportional to the RNA level of corresponding gene although, as shown by Held et al. in 2006 the relationship is not linear whether or not the data are pre-processed.*

Finally, here are few minor comments.

*Authors’ response: Minor comments of the reviewer have been taken into account. Corresponding changes in the manuscript are highlighted in grey.*

1. Introduction, line 2. “physical or chemical conditions”. Perhaps biological conditions too?

2. Introduction, line 4. It seems like “their” should be inserted between “evaluate” and “variability”.

3. Introduction, paragraph 2, sentence 2. Aren’t “specific methods used” and “inaccuracies of the measurements” themselves “experimental factors”?

4. Introduction, last paragraph. What is the difference between “accuracy” and “precision”? Also, how is specificity defined in the case of microarrays?

*Author’s response: Definitions of accuracy and precision can be found in ref. 45.*

*In the case of microarrays specificity refers to the ability of a probe to bind a unique target sequence. A specific probe provides signal proportional only to the amount of the target sequence, while non-specific probe signal will be a result of interaction with more than one target sequence. The specificity of a probe can be diminished by cross-hybridization, also called non-specific hybridization, a phenomenon in which sequences that are not strictly complementary according to the Watson–Crick rules bind to each other.*

5. Introduction, last sentence. Delete “have”.

6. Microarray Structure, paragraph 2. “…600 nt sequence fragments located near the 3′ end of a specific transcript (Fig. 1). “This is not clear from Fig. 1.

7. Microarray Structure, last paragraph. Platform differences contribute to low accuracy of microarrays, so “despite” seems to be out of place.

8. Step I: RNA isolation, paragraph 1. “…ribosomal RNA… is rarely studied”. I think rRNA is studied quite extensively in phylogeny and pharmacology.

9. Step I: cDNA synthesis, paragraph 1. Shouldn’t the figure referred to here be 4 rather than 3?

10. Conclusions, paragraph 1. What is the significance of “morphological features” in this context?

11. Caption to Fig. 1. What is the purpose of probe sets A and B? Also, does “corresponds to” mean “proportional to”, see technical comments 4?

12. Figure 2. I think it would be better if the steps of microarray experiment shown in the figure correspond to the steps described under Biological background of microarray experiments.

13. Table 2. What about the other two combinations of control probe-set outcomes involving an error?

14. The authors are encouraged to proofread their submission. There are a few places with missing or extra commas, instances where article “the” can (or perhaps should) be removed, etc.

### Reviewer’s report 3: Dr. I King Jordan

This reviewer provided no comments for publication.

## Abbreviations

3′IVT: 3′ in vitro transcription
3′-UTR: 3′ untranslated region
BGP: Background Intensity Probes
ChIP: Chromatin immunoprecipitation
cDNA: Complementary DNA
ERCs: External RNA controls
MM: Mismatch
PCR: Polymerase chain reaction
PM: Perfect match
rRNA: Ribosomal RNA
SNP: Single-nucleotide polymorphism
